# Cytoplasmic Localization of HTLV-1 HBZ Protein: A Biomarker of HTLV-1-Associated Myelopathy/Tropical Spastic Paraparesis (HAM/TSP)

**DOI:** 10.1371/journal.pntd.0005285

**Published:** 2017-01-17

**Authors:** Marco Baratella, Greta Forlani, Goutham U. Raval, Alessandra Tedeschi, Olivier Gout, Antoine Gessain, Giovanna Tosi, Roberto S. Accolla

**Affiliations:** 1 Department of Surgical and Morphological Sciences, School of Medicine, University of Insubria, Varese, Italy; 2 Service de Neurologie, Fondation Ophtalmologique Adolphe de Rothschild, Paris, France; 3 Unité d'Epidémiologie et Physiopathologie des Virus Oncogènes, Institut Pasteur and Centre National de la Recherche Scientifique, Paris, France; George Mason University, UNITED STATES

## Abstract

HTLV-1 is the causative agent of a severe form of adult T cell leukemia/Lymphoma (ATL), and of a chronic progressive neuromyelopathy designated HTLV-1 associated myelopathy/tropical spastic paraparesis (HAM/TSP). Two important HTLV-1-encoded proteins, Tax-1 and HBZ, play crucial roles in the generation and maintenance of the oncogenic process. Less information is instead available on the molecular and cellular mechanisms leading to HAM/TSP. More importantly, no single specific biomarker has been described that unambiguously define the status of HAM/TSP. Here we report for the first time the finding that HBZ, described until now as an exclusive nuclear protein both in chronically infected and in ATL cells, is instead exclusively localized in the cytoplasm of peripheral blood mononuclear cells (PBMC) from patients suffering of HAM/TSP. Interestingly, at the single cell level, HBZ and Tax-1 proteins are never found co-expressed in the same cell, suggesting the existence of mechanisms of expression uncoupling of these two important HTLV-1 viral products in HAM/TSP patients. Cells expressing cytoplasmic HBZ were almost exclusively found in the CD4+ T cell compartment that was not, at least in a representative HAM/TSP patient, expressing the CD25 marker. Less than 1 percent CD8+ T cells were fond positive for HBZ, while B cells and NK cells were found negative for HBZ in HAM/TSP patients. Our results identify the cytoplasmic localization of HBZ in HAM/TSP patient as a possible biomarker of this rather neglected tropical disease, and raise important hypotheses on the role of HBZ in the pathogenesis of the neuromyelopathy associated to HTLV-1 infection.

## Author Summary

Currently, more than 10 million people worldwide are infected with HTLV-1, the first discovered human oncogenic retrovirus. Up to 7% of infected individuals experience during their life a severe form of T cell malignancy or a chronic progressive inflammatory disease of the nervous system designated HTLV-1-associated myelopathy/tropical spastic paraparesis (HAM/TSP). At present, there is no resolutive therapy for both of these diseases. In HAM/TSP patients, besides classical neurological signs and the degree of proviral load, no specific virus-related biomarker has been defined that unambiguously distinguishes infected cells of HAM/TSP from those of asymptomatic carriers or ATL patients. Here for the first time, we present evidence that an HTLV-1 protein, designated HBZ, previously found expressed only in the nucleus, is indeed exclusively localized in the cytoplasm of peripheral blood mononuclear cells of HAM/TSP patients and almost exclusively in the CD4+ T cell compartment without the need that these cells co-express the Treg-associated marker CD25. This finding establishes an association between development of the inflammatory HAM/TSP disease and presence of a viral product in the cytoplasm, opening new ways to understand the molecular basis of the HTLV-1-mediated pathogenesis of this severe form of neuromyelopathy.

## Introduction

HTLV-1 is an oncogenic human retrovirus whose infection affects at least ten million people worldwide [1]. HTLV-1 is the pathogenic agent of a severe form of leukemia/lymphoma designated Adult T-cell Leukemia/Lymphoma (ATL) characterized by the malignant transformation of CD4+ T cells [2] and of a severe neurological disorder designated HTLV-1-associated myelopathy/tropical spastic paraparesis (HAM/TSP), a chronic progressive neuromyelopathy characterized by spastic paraparesis, sensory dysfunction and sphincter function defects [3, 4]. Like other retroviruses HTLV-1 produces structural proteins, Gag, Pol and Env, encoded by the plus strand of the viral genome [5]. The HTLV-1 genome expression is mainly influenced by two regulatory proteins, Tax-1 and Rex, encoded by the 3’ region of viral genome between *env* and 3’ LTR [5]. The viral protein Tax-1 is important for the transcription of the provirus and its oncogenic potential [6, 7]. The minus strand of the viral genome encodes a transcript whose protein product is designated HTLV-1 bZIP factor (HBZ) [8]. It is of note that, while Tax-1 is expressed only in 40% of cells from ATL patients, HBZ transcripts are constantly found in all ATL cells [5, 9, 10]. This reflects the fact that HBZ is also important for infectivity and persistence *in vivo* [11]. HBZ contains a bZIP domain, an activation (N-terminus) and a central domain [8]. There are two different isoforms of this protein: a spliced form containing 206 amino acids and an unspliced form with 209 amino acids, leading to proteins that differ in only seven amino acids at their N-terminal AD domains [12, 13]. The spliced form is more abundant than the unspliced form and is found in almost all ATL patients [14]. HBZ has been described as a nuclear protein, appearing in speckle-like structures [15]. The nuclear localization has been correlated with the presence of several nuclear localization signals present in the basic regions and in the DNA binding domain of the molecule [16]. Localization studies, however have been performed in cell lines often not representative of the real targets of HTVL-1 infection and over-expressing HBZ after transfection with the encoding gene. Experiments using cells transfected with tagged HBZ have shown that HBZ interacts with CREB-2 via its bZIP domain resulting in strong inhibition of the CREB-2/Tax-1 interaction instrumental for the activation of HTLV-1 LTR [8]. Beside the inhibitory effect on the transcription of the viral genome, a series of studies have indicated that HBZ has the capacity to interact with a large array of cellular transcription factors modulating in a positive or negative fashion their biological activity on cell homeostasis [17]. Again, most of these studies have been performed in HBZ-transfected cells raising the possibility that the results obtained might bear limited biochemical and functional relevance. Only recently, by the use of the first reported monoclonal antibody against HBZ, 4D4-F3, generated in our laboratory, it was possible to investigate for the first time the expression and the biochemical interaction with host factors of endogenous HBZ in HTLV-1 chronically infected cells, in ATL cell lines and, most importantly in fresh PBMCs of an ATL patient [18]. Endogenous HBZ is indeed expressed in speckle-like structures localized in the nucleus, although deprived of aggregates usually seen in HBZ-transfected cells [8, 19]. A careful quantification of endogenous HBZ let us to show that the viral protein is expressed in the range of 18.000–40.000 molecules per cell, 20–50 fold less than the amount expressed in HBZ-transfected cells. In chronically infected cells and in ATL, HBZ interacts *in vivo* with p300 and JunD and co-localizes only partially, and depending on the amount of expressed HBZ, not only with p300 and JunD but also with CBP and CREB2 [18].

The present investigation was set to determine whether we could detect and define by confocal microscopy the subcellular localization of endogenous HBZ in the other major HTLV-1-associated pathology, HAM/TSP. Moreover, the analysis was extended to other cases of ATL and also to infected asymptomatic carriers (AC). Interestingly, and unexpectedly, the analysis of PBMCs from four distinct HAM/TSP patients unequivocally showed that HBZ protein is exclusively localized in the cytoplasm. Nuclear localization of HBZ was instead confirmed in the vast majority of fresh PBMC of ATL patients, whereas we were unable to detect HBZ in cells of AC. In HAM/TSP, the percentage of HBZ-positive PBMC cells ranged between 0,4% and 11%. Tax-1 was detected in PBMC of three out of four of these patients, at percentages ranging between 1 to 20%. Interestingly, no co-expression of HBZ and Tax-1 was found in the same cell. Tax-1 was not detected in cells of ATL patients.

These results are discussed within the frame of the present knowledge of the pathogenesis of HTLV-1-associated diseases.

## Results

### Cytoplasmic localization of HTLV-1 HBZ protein in PBMC of HAM/TSP patients

**Fig 1:**
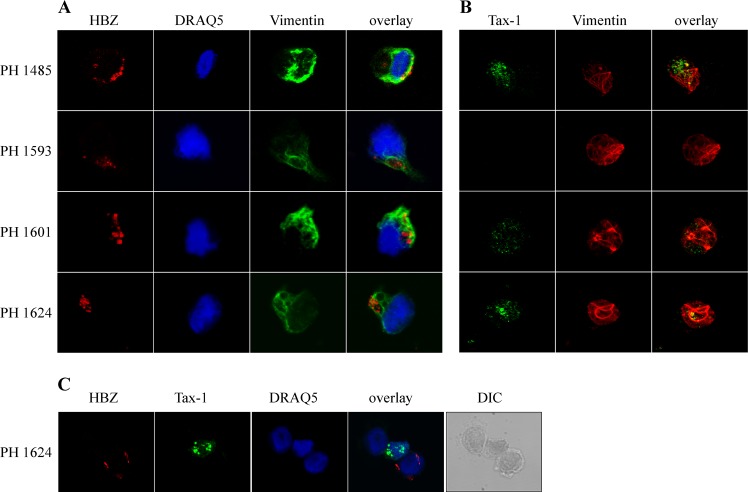
Subcellular localization of endogenous HBZ and Tax-1 in PBMC of HAM/TSP patients. (A) PBMC of four HAM/TSP patients (PH1485, PH1593, PH1601, and PH1624) were stained with the 4D4-F3 anti-HBZ mAb followed by Alexa Fluor 546-conjugated goat anti-mouse IgG1 antibody (red) and (B) with the A51-2 anti-Tax-1 mAb followed by Alexa Fluor 488-conjugated goat-anti-mouse IgG2a antibody (green), and analyzed by confocal microscopy. Specific counterstaining of nucleus or cytoplasmic compartments was performed by using DRAQ5 fluorescence probe to detect the nucleus and anti-vimentin rabbit polyclonal antibody followed by goat anti-rabbit IgG conjugated to Alexa Fluor 488 (green, panel A) or to Alexa Fluor 546 (red, panel B) to stain the cytoplasmic compartment. At least 300 cells were analyzed; a representative cell for HBZ or for Tax-1 staining is shown for each patient.(C) Low magnification field to show the co-existence of cells mutually exclusive for the expression of cytoplasmic HBZ and Tax-1 in PBMC of patient PH1624. Cells were co-stained with 4D4-F3 anti-HBZ mAb and with A51-2 anti-Tax-1 mAb followed by specific secondary antibodies staining as described in (A) and (B).

In order to define the subcellular localization of endogenous HBZ in HAM/TSP, PBMC of four HAM/TSP patients, namely PH1485, PH1593, PH1601 and PH1624 were studied by immunofluorescence and confocal microscopy analysis. In striking contrast with the HBZ nuclear localization in ATL [18], in all four HAM/TSP patients HBZ localization was confined to the cytoplasm (Fig 1A). Parallel staining with DRAQ5, a nuclear marker, and with vimentin, a cytoplasmic marker, confirmed the exclusive HBZ cytoplasmic localization. HBZ appeared as discrete dots, similar in shape to the nuclear speckles found in PBMC of ATL patients. The percentage of HBZ positive cells was comprised between 4 to 11% of total PBMC in three of the four patients analyzed with the exception of patient PH1601 having only 0,4% HBZ-positive cells (Table 1). PBMC from HAM/TSP patients were then analyzed for the expression and subcellular localization of Tax-1 protein. Tax-1 was expressed in 1%, 14% and 20% of the PBMC of PH1601, PH1485 and PH1624 patients, respectively (Table 1). Tax-1 was undetectable in PH1593 PBMC. In patient PH1485 Tax-1 was localized in dot-like structures in the nucleus **(**Fig 1B**).** A proportion of these cells expressed Tax-1 also in the cytoplasm. In patient PH1624, Tax-1 was localized in the nucleus with a large proportion of these cells expressing Tax-1 also in the cytoplasm. Importantly, no cells were found to co-express HBZ and Tax-1, as documented in Fig 1C for the PBMC of patient PH1624 displaying a high percentage of both HBZ+ (9%) and Tax-1+ (20%) cells (see also Table 1).

**Table 1:** Percent and subcellular distribution of HBZ+ and Tax-1+ PBMC in HTLV-1-associated pathologies.

Patient	Ab titer*	Pathology	HBZ+ cells (%)			Tax-1+ cells (%)		
			Total	Nucleus	Cyto	Total	Nucleus	Cyto
**PH1485**	**640**	**HAM/TSP**	**11**	**0**	**11**	**14**	**14**	**5^**
**PH1593**	**2560**	**HAM/TSP**	**4**	**0**	**4**	**0**	**-**	**-**
**PH1601**	**2560**	**HAM/TSP**	**0,4**	**0**	**0,4**	**1**	**1**	**0**
**PH1624**	**2560**	**HAM/TSP**	**9**	**0**	**9**	**20**	**20**	**12^**
**PH1393**	**320**	**ATL**	**83**	**83**	**0**	**0**	**-**	**-**
**PH1505**	**1280**	**ATL**	**80**	**83**	**0**	**0**	**-**	**-**
**PH1614**	**640**	**AC**	**0**	**-**	**-**	**11**	**11**	**5^**
**PH1619**	**640**	**AC**	**0**	**-**	**-**	**1**	**1**	**0**
**PH1621**	**1024**	**AC**	**0**	**-**	**-**	**6**	**6**	**2^**

* reverse dilution values of anti-HTLV-1 antibody defined as described in Materials and Methods.

^ percent of cells with Tax-1 localization in cytoplasm and nucleus. Tax-1 was never localized in cytoplasm alone

**Fig 2:**
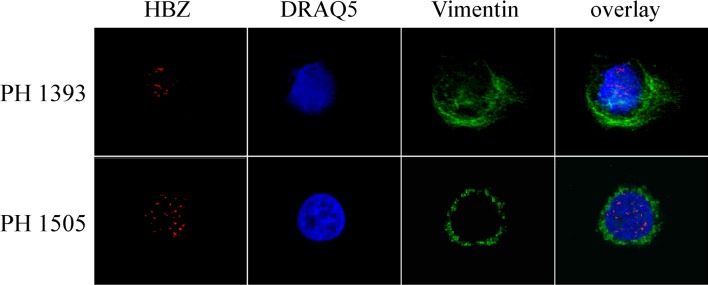
Subcellular localization of endogenous HBZ and Tax-1 in PBMC of ATL patients. PBMC of two ATL patients (PH1393 and PH1505) were stained with the anti-HBZ 4D4-F3 mAb, followed by Alexa Fluor 546-conjugated goat anti-mouse IgG1antibody to detect the HBZ protein and analyzed by confocal microscopy. Specific counterstaining of nucleus or cytoplasmic compartments was performed by using DRAQ5 fluorescence probe or anti-vimentin antibody as describe in the legend to Fig 1. At least 300 cells were analyzed; a representative cell for HBZ staining is shown for each patient. PBMC from both patients were negative for expression of Tax-1 protein.

To expand our previous studies in ATL patients, PBMC of two additional patients, PH1393 and PH1505, were investigated by immunofluorescence and confocal microscopy. In PBMC of both patients, 80% and 83% of the cells were positive for HBZ (Table 1). HBZ was localized in speckle-like structures in the nucleus **(**Fig 2**)** in a very similar fashion as in the previously described PH961 patient [18]. Parallel staining for the nuclear marker DRAQ5, and for the cytoplasmic marker vimentin, confirmed the exclusive nuclear localization of HBZ. PBMC of the two ATL patients were also analyzed for the presence of Tax-1 protein; none of them showed positivity for this viral protein (Table 1).

**Fig 3:**
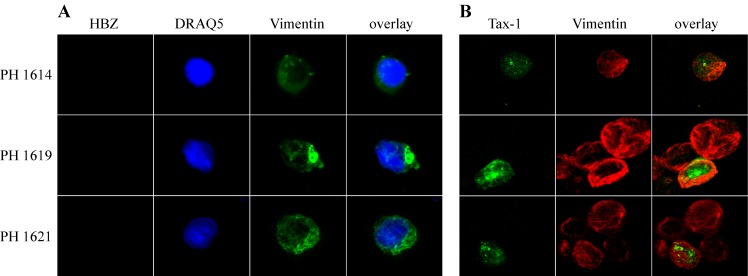
Lack of endogenous HBZ, but not of Tax-1, detection in PBMC of HTLV-1 positive asymptomatic carriers. (A) PBMC of three HTLV-1-positive asymptomatic carriers (PH1614, PH1619, and PH1621) were stained with the anti-HBZ 4D4-F3 and (B) the anti-Tax-1 A51-2 mAbs, followed by Alexa Fluor 546-conjugated goat anti-mouse IgG1antibody to detect HBZ protein (red), or by Alexa Fluor 488-conjugated goat-anti-mouse IgG2a to detect Tax-1 (green), and analyzed by confocal microscopy. Specific counterstaining of nucleus or cytoplasmic compartments was performed by using DRAQ5 fluorescence probe to detect the nucleus and anti-vimentin rabbit polyclonal antibody followed by goat anti-rabbit IgG conjugated to Alexa Fluor 488 (green, panel A) or to Alexa Fluor 546 (red, panel B) to stain the cytoplasmic compartment. At least 300 cells were analyzed; a representative cell is shown for each patient.

Interestingly, PBMC from the three asymptomatic carriers analyzed, PH1614, PH1619 and PH1621, did not show any positivity for HBZ **(**Fig 3A), although they expressed Tax-1 in a small but distinctive proportion of cells (11%, 1% and 6%, respectively, Table 1) with a preferential, although not exclusive, nuclear localization **(**Fig 3B**).** Taken together, these results establish for the first time a striking difference in the subcellular localization of HBZ protein in HAM/TSP patients as compared to ATL patients, showing the unprecedented exclusive cytoplasmic localization of HBZ in HAM/TSP patients.

### HBZ in HAM/TSP stably resides in the cytoplasm and does not shuttle to and from the nucleus

**Fig 4:**
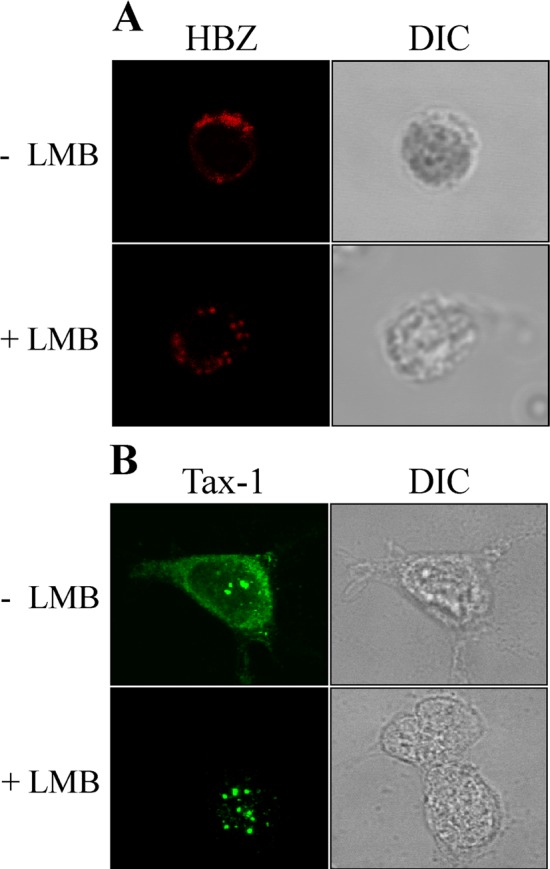
HBZ is a resident cytoplasmic protein and does not shuttle to the nucleus in HAM/TSP. (A) PBMC of HAM/TSP patient PH1624 were either treated (+ LMB) or untreated (- LMB) with LMB, an inhibitor of nuclear export, before fixing and staining with the anti-HBZ 4D4-F3 mAb followed by Alexa Fluor 546-conjugated goat anti-mouse IgG1antibody (red). (B) As control of inhibition of nuclear export by LMB, 293T cells transfected with the plasmid coding for Tax-1 viral protein were treated with LMB (+LMB) or left untreated (-LMB). Cells were fixed and stained with A51-2 anti Tax-1 mAb followed by Alexa Fluor 488-conjugated goat-anti-mouse IgG2a (green), and analyzed by confocal microscopy. DIC represents the differential interference contrast image.

In order to assess whether the cytoplasmic localization of HBZ in PBMC of HAM/TSP patients is a stable feature or dynamic event resulting from a rapid recycling of the protein from the nucleus, PBMC from PH1624 patient were treated with Leptomycin B (LMB), an inhibitor of nuclear export, and analyzed by immunofluorescence and confocal microscopy. Results clearly indicate that the HBZ cytoplasmic localization in PBMC of HAM/TSP is not affected by LMB treatment **(**Fig 4A). Conversely, as we previously showed [20] this treatment resulted in a prominent nuclear retention of Tax-1 protein in 293T cells transfected with a Tax-1-encoding cDNA **(**Fig 4B**).** From these results we conclude that in HAM/TSP patients, HBZ is specifically retained into the cytoplasm and does not shuttle into the nucleus.

### HBZ-positive cells in HAM/TSP representative PH1624 patient are found in the CD4+/CD25- T cell subpopulation

**Fig 5:**
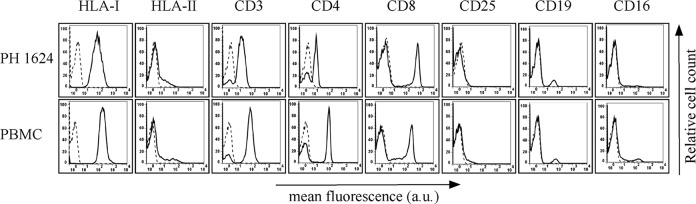
Expression of cells surface markers in PBMC of HAM/TSP patient PH1624 and healthy control. The expression of HLA class I, HLA class II DR, CD3, CD4, CD8, CD25, CD19, and CD16 surface molecules on PBMC from TSP/HAM patient PH1624 and a healthy control was assessed by immunofluorescence and flow cytometry with antibodies specific for the various markers. Results are expressed as relative number of cells (ordinate) versus the mean intensity of fluorescence in arbitrary units (abscissa). In each histogram, negative controls, obtained by staining the cells with an appropriate isotype-matched antibody, are depicted as dashed line.

In order to define the cellular subpopulation expressing the cytoplasmic HBZ protein in HAM/TSP patients, we analyzed in detail the PBMC of patient PH1624 displaying one of the highest number of HBZ-positive cells (9%). Initial analysis of relevant cell surface markers by immunofluorescence and flow cytometry (Fig 5 and Table 2) showed that this patient expressed CD3, CD4 and CD8 markers in 88%, 63% and 27% of PBMC, respectively. This phenotype was very similar to the one of PBMC from a normal donor. Interestingly the T cell marker CD25, known to be expressed in activated as well as in regulatory T (Treg) cells was not detected in PH1624 PBMC, whereas was expressed at low amount in 4–5% of normal PBMC. The B cell compartment, as assessed by the presence the CD19 marker, was represented in almost equal proportion of PH1624 (10%) and normal (8%) PBMC. NK cells, as assessed by the CD16 marker, were 4% of PH1624 PBMC and 10% of normal PBMC. HLA class I was expressed in 100% of both PH1624 and normal PBMCs, and HLA class II was expressed in 15% and 18% of PH1485 and normal PBMC, respectively.

**Table 2:** Percent of HBZ-positive cells in PBMC subpopulations of HAM/TSP PH1624 patient.

CD4 (63%)*	CD8 (27%)	CD25 (0%)	CD19 (10%)	CD16 (4%)
**15^**	**< 1**	**0**	**0**	**0**

*values in parenthesis represent the percentage of positive cells with respect to the total PBMC population analyzed, as assessed by immunofluorescence and FACS analysis.

^percent of HBZ+ cells within each specific subpopulation.

**Fig 6:**
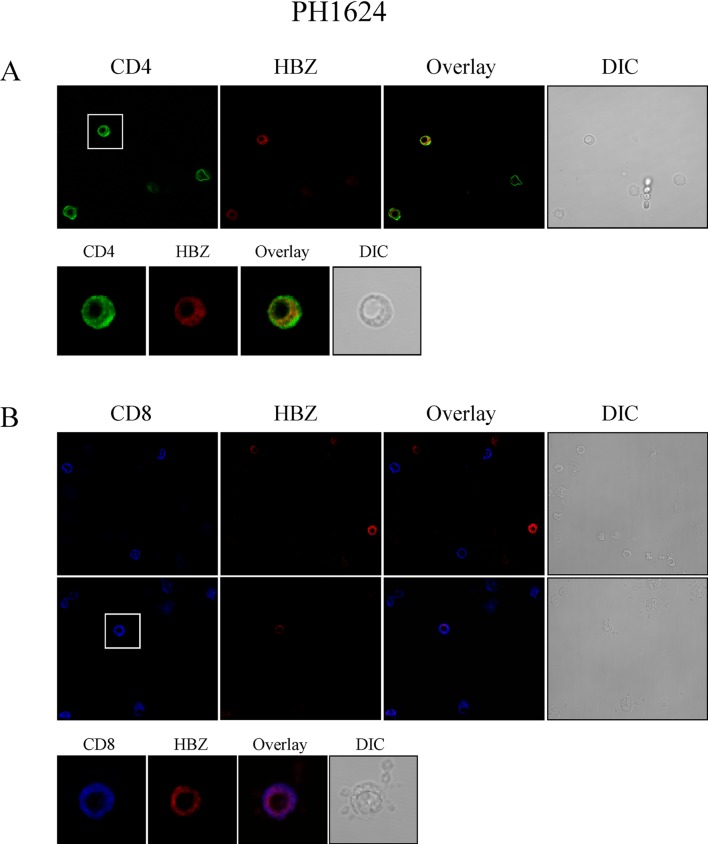
HBZ is preferentially expressed in CD4+ T cells of HAM/TSP patient PH1624. Confocal microscopy analysis of PBMC from HAM/TSP patient PH1624. (A) co-staining with the 4D4-F3 anti-HBZ mAb followed by Alexa Fluor 546-conjugated goat anti-mouse IgG1 antibody (red) and with the anti-CD4 mAb followed by Alexa Fluor 488-conjugated goat-anti-rabbit IgG antibody (green); upper panels, extended field; lower panels, enlarged field focused on the single cell depicted in the square of the left upper panel and positive for both CD4 and HBZ. (B) co-staining with the 4D4-F3 anti-HBZ mAb followed by Alexa Fluor 546-conjugated goat anti-mouse IgG1 antibody (red) and with the anti-CD8 rabbit monoclonal antibody directly conjugated to Alexa Fluor 647 (blue); upper panels depict extended fields with CD8+ cells negative for HBZ; middle panels, extended fields with many CD8+ cells negative for HBZ and the single CD8+/HBZ+ cell (square); lower panels, enlarged field on the single CD8+/HBZ+ cell.

**Fig 7:**
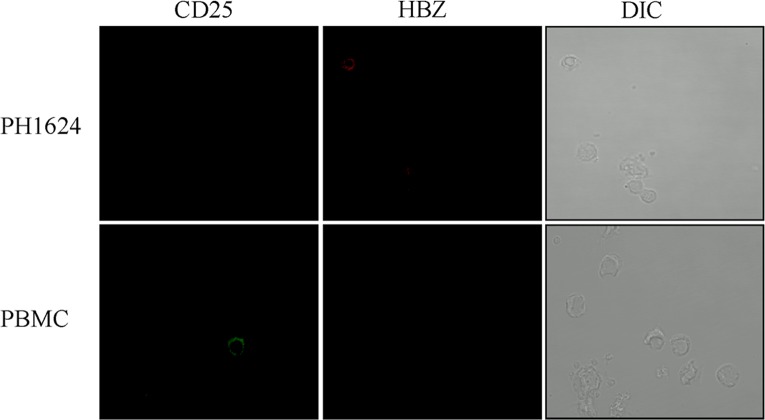
Lack of CD25+ cell in PBMC of patient PH1624, as assessed by confocal microscopy. PBMC of HAM/TSP patient PH1624 and of healthy control were co-stained with the anti-HBZ 4D4-F3 mAb followed by Alexa Fluor 546-conjugated goat anti-mouse IgG1 antibody (red) and with the anti-CD25 monoclonal antibody directly conjugated to Alexa Fluor 488 (green) and analyzed by confocal microscopy. Upper left panel shows the negativity of CD25 staining in PH1624 patient as compared with a positive cell for the same marker in PBMC of healthy control (PBMC, lower right panel)

Subsequently, confocal microscopy analysis was performed. Cytoplasmic HBZ was clearly detected in a significant proportion of CD4+ cells (Fig 6A, extended field, top panels, and focus on single cell, bottom panels). Indeed around 15% of CD4+ T cells were also HBZ+ (Table 2). Considering that cytoplasmic HBZ was detected in 9% of the total PBMC of PH1624 patient, and that CD4+ T cell represented around 63% of the PBMC in this patient, it derives that virtually all HBZ+ cells are included in CD4+ T cell compartment. Indeed cytoplasmic HBZ+ cells were virtually not detected in the CD8+ PBMC of PH1624 patient (Fig 6B, top panels, extended fields). After careful analysis of more than 100 CD8+ T cells, in fact only one cell was found to co-express HBZ in its cytoplasm (Fig 6B, middle panels, extended field, and focus on the single positive cell, lower panel). Confocal analysis of CD25 expression in PH1624 patient’s PBMC confirmed that cells were negative for this marker (Fig 7, upper left panel). Similarly, neither CD19+ B cells nor CD16+ NK cells were found to express cytoplasmic HBZ (Table 2). Thus, in a representative HAM/TSP patient displaying a high percentage of HBZ-positive cells, cytoplasmic HBZ is almost exclusively found in CD4^+^ T cells and these cells do not co-express the CD25 marker.

## Discussion

Infection by HTLV-1 generally leads to a state of asymptomatic carrier which may last for the entire life. However, in 3–7% of individuals a very severe form of leukemia/lymphoma, ATL, or a chronic progressive neurological disease can develop as result of the infection. Other less frequent diseases are also associated causally with HTLV-1 infection in certain high endemic areas as uveitis in Japan and infective dermatitis in South America, Africa and the Caribbean. The oncogenic progress leading to ATL has been mainly attributed to the viral transactivator Tax-1 that hijacks the basic mechanisms of control of cellular homeostasis [21]. Nevertheless, only 40% of ATL patients can express Tax-1 whereas all of them express HBZ, an event that has been interpreted as an involvement of Tax-1 in the first phases of oncogenic process and of HBZ in the maintaining of leukemic state [11, 22]. While recent data have unambiguously demonstrated the nuclear localization of endogenous HBZ in ATL cells and in chronically infected cell lines [18], no data were available on the endogenous expression and subcellular localization of HBZ in patients affected by HAM/TSP. In the present study we demonstrate that a discrete percentage, up to 11%, of PBMC from four HAM/TSP patients express HBZ and that this expression is exclusively confined to the cytoplasm. Cytoplasmic HBZ appears distributed in dots similar to the nuclear speckle-like structures observed in leukemic cells of ATL patients ([18] and this study), sometimes dispersed all over the cytoplasm, sometimes concentrated in a restricted area of it.

The relatively low percentage of cells expressing HBZ in otherwise numerically normal PBMC from HAM/TSP patients, and the availability of only small samples of PBMC from the patients under study prevented a biochemical analysis of the molecular basis of HBZ retention in the cytoplasm. Future studies will be concentrated toward this crucial aspect, in conjunction with a more refined characterization of the sub-cytoplasmic compartments where HBZ is located. Within this frame, leukapheresis in selected patients to obtain larger number of cells will certainly be needed for both biochemical and confocal analyses at subcellular level. Nevertheless, several hypotheses can be put forward to help explaining our findings and to orient future studies. For example, two distinct forms of HBZ derived from distinct mRNAs, spliced and unspliced, have been described [12–14]. Spliced and unspliced HBZ display more than 95% sequence homology and diverge only in the first seven N-terminal aminoacids. Unspliced and spliced HBZ should be both present in HAM/TSP and have been found at least at level of mRNA [23]. Although previous studies have suggested that both unspliced and spliced HBZ localize into the nucleus [13, 15], particularly in ATL cells, the alternative possibility that one of the two HBZ forms may preferentially distribute in the cytoplasmic region in HAM/TSP cannot be excluded. The 4D4-F3 anti-HBZ monoclonal antibody used in this study was raised against the spliced form of the protein; however, the epitope recognized by the antibody should be present in both spliced and unspliced HBZ, as it maps within the BR1 region, between aa 97–135 [18]. Thus, the 4D4-F3 mAb may not be the appropriate tool to solve this issue. It has been recently described the existence of aminoacid variations in the HBZ protein both in asymptomatic carriers and HAM/TSP patients. These variations were identified in the activation domain and in the nuclear localization signal sequence [24]. Thus, it might be possible that HBZ sequence variation may influence the subcellular localization of the protein. In this regard, it is important to underline that cytoplasmic localization of HBZ in PBMC of HAM/TSP patients was not modified by the treatment of the cells with Leptomycin B, a drug that blocks the CRM1-dependent nuclear-cytoplasmic shuttling of the proteins, strongly indicating that HBZ is a cytoplasmic resident, non migrating protein in HAM/TSP. It should be underlined that we were unable to detect HBZ-positive cells in PBMC of the asymptomatic carriers analyzed in this study. This could be due to the level of HBZ expression under the threshold of detectability of our method or to the real absence of HBZ expression in AC. Compartmentalization of HBZ in specific subcellular cytoplasmic and/or nuclear structures not detectable by our antibody appears unlikely, although cannot be completely excluded *a priori*. Future studies will clarify this aspect.

An additional interesting finding reported in the present study concerns the expression and localization of HTLV-1 Tax-1 at the single cell level. In PBMC of HAM/TSP patients, when detectable, Tax-1 was localized always in the nucleus and in a variable proportion of the cells in both nucleus and cytoplasm. Importantly, it was never expressed in the same cells expressing HBZ. While uncoupling of Tax-1 and HBZ expression is rather common in ATL patients [5, 9, 10, 14], the mutually exclusive expression of either HBZ or Tax-1 proteins at the single cell level has never been reported. The molecular basis of this event in cells of HAM/TSP patients is unknown at present, and certainly will be the focus of our future investigation. It is tempting to speculate that this finding may be relevant at the functional level and particularly in the immune recognition of HTLV-1 infected cells. The HBZ cytoplasmic localization, possibly in extra-endosolic compartments, may not be appropriate to the generation of peptides that can efficiently bind MHC class I molecules for presentation to, and scrutiny by cytotoxic T cells (CTLs). This may explain the relatively low level of HBZ-specific CTLs in HAM/TSP [25, 26] in conjunction with the unsatisfactory lytic efficiency of HBZ-specific CTLs with respect to the strong recognition and lytic efficiency of Tax-1-specific CTLs [27].

Additional confocal microscopy analysis of PBMC of a representative HAM/TSP patient PH1624 clearly showed that the major, if not the exclusive, HBZ+ cell subpopulation was represented by CD4+ cells. Indeed around 15% of CD4+ cells expressed cytoplasmic HBZ. Conversely, after counting a large number (more than 100 cells) of CD8+ PH1624 cells, only 1 was found to co-express cytoplasmic HBZ. Thus, if from one side this result indicates that CD8+ cells can be infected by HTLV-1 and express HBZ in the HAM/TSP patients, this event is extremely rare as compared to the frequency of HBZ+/CD4+ cells. Absence of HBZ protein expression was also demonstrated in B cells and in NK cells. PBMC of HAM/TSP patient PH1624 did not reveal the presence of CD25+ T cells both by FACS and by confocal analyses. Further refinement by confocal analysis confirmed the absence of CD4+/CD25+ T cells, a subset that includes regulatory T cells (Treg). Although CD4+/CD25+ Tregs can be infected by HTLV-1 and HAM/TSP patients have been shown to have a high number of CD4+/CD25+ Tregs with impaired function (reviewed in [28]) as well as HBZ mRNA expression levels comparable to those observed in ATL [29], the results presented in this paper indicate that in HAM/TSP patients HBZ protein expression can be easily found in CD4+ T cells not displaying the classical phenotype of Treg cells. Additional studies are certainly required to further detail the phenotype and the functional correlate of CD4+ T cells expressing HBZ protein in HAM/TSP patients as well as the possibility that other cells infected by HTLV-1, such as monocytes/macrophages, may express HBZ protein in their cytoplasm.

In conclusion, based on the results presented in this paper we propose that HBZ cytoplasmic localization can be considered as a *bona fide* biomarker of HTLV-1-derived HAM/TSP pathology. Future studies will be addressed to the assessment of cytoplasmic HBZ localization during HTLV-1 infection and in the follow-up of infected people before they acquire clear signs of pathology, to possibly identify the cytoplasmic HBZ localization not only as a biomarker but also as a predictive element of HAM/TSP development.

## Materials and Methods

### Ethics statement

We obtained PBMCs from HTLV-1 asymptomatic donors, HAM/TSP patients and ATL patients in the context of a Biomedical Research Program approved by the Committee for the Protection of Persons, Ile-de-France II, Paris (2012-10-04 SC). All individuals gave informed consent. Patient’s data were analyzed anonymously.

### Cells

Human embryonic kidney 293T cells (kindly provided by Prof. B.M. Peterlin, UCSF, San Francisco, USA) were cultured in Dulbecco’s modified Eagle medium (DMEM) containing 5 mM L-glutamine and supplemented with 10% fetal calf serum (FCS). Peripheral blood monuclear cells (PBMC) from healthy donors, HTLV-1+ asymptomatic carriers, HAM/TSP patients were purified by Ficoll-Paque TM PLUS (GE-Healthcare Bio-Science, Milan, Italy) of heparinated blood. PBMC from healthy donors were obtained by the Blood Transfusion Center, Ospedale di Circolo, Fondazione Macchi, Varese, whereas PBMC of HTLV-1-infected patients were obtained through the Biomedical Research Program approved by the Committee for the Protection of Persons, Ile-de-France II, Paris (2012-10-04 SC). All PBMC preparations were immediately frozen at -80°C and subsequently transferred in liquid nitrogen after 48–96 hours. HTLV-1 infection was confirmed by Western blot on plasma sample and anti-HTLV-1 antibodies in patient’ plasma were titrated through indirect immunofluorescence using the HTLV-1-producing MT-2 cell line (kindly provided by Dr. B. Macchi, Tor Vergata University, Rome, Italy), as described previously [30]. The two ATL patients analyzed in this study, PH1393 and PH1505, were suffering from a typical acute ATL form characterized by high hyperlymphocytosis with lymphocyte count of 18.000/mm^3^ and 30.000/mm^3^, respectively, and with 79% and 80% of atypic lymphocytes, as evaluated by optical microscopy.

### Transfection and treatments

293T cells cultured on glass coverslips pre-coated with poly-L-lysine were transfected with 0.2 μg of plasmid expressing untagged Tax-1 by using FuGENE HD (3 μl/μg DNA; Promega, Milan, Italy) as described previously [20]. Where indicated, the 293T cells or HAM/TSP PBMC were incubated with 20 nM leptomycin B (LMB; Sigma) or the vehicle methanol for 3 h at 37°C, 5% CO2.

### Immunofluorescence and confocal microscopy

Frozen vials containing PBMC were thawed by immediate passage from liquid nitrogen to a water bath set at 37°C. Cells were washed with warm RPMI medium and immediately processed for immunofluorescence and flow cytometry analysis or for confocal microscopy, as described [31]. For flow cytometry, the following reagents were used: mouse anti-human HLA class I (clone B9.12); mouse anti-human HLA class II DR (clone D1.12), both revealed by FITC-labelled rabbit anti-mouse IgG F(ab’)2 antiserum (Sigma, Milan, Italy); FITC mouse anti-human CD3 (clone (UCHT1, BD Pharmingen); FITC mouse anti-human CD4 (clone RPA-T4, BD Pharmingen); PE-Cy5 mouse anti-human CD8a (clone RPA-T8; eBioscience, Milan, Italy); PE mouse anti-human CD16 (clone B73.1, eBioscience, Milan, Italy); FITC mouse anti-human CD19 (clone HIB19, BD Pharmingen) and phyco-erythrin (PE) mouse anti-human CD25 (clone M-A251, BD Pharmingen).

For confocal microscopy, appropriate number of cells were cultured on glass coverslips pre-coated with poly-L-lysine (0.1gr/ml, Sigma) for five hours. The cells were then washed with 1x PHEM buffer, pH 6.9 (60 mM PIPES, 25 mM HEPES, 10mM EGTA, 2mM MgCl2) three times, fixed in methanol 7 minutes at -20°C, and blocked with 1% BSA in 1x PHEM for 1h at room temperature (RT). Cells were then stained overnight with anti-HBZ 4D4-F3 monoclonal antibody (mAb), anti-Tax-1 mAb (clone 168 A51-2 from the NIH AIDS Research and Reference Reagent Program), anti-vimentin rabbit polyclonal antibody (Santa Cruz Biotechnology, CA, USA), rabbit anti-CD4 monoclonal antibody (clone EPR6855, ABCAM) and anti-CD19 rabbit monoclonal antibody (clone EPR5906, ABCAM), diluted in PHEM buffer containing 0.5% BSA. The slides were then washed five times with cold 1x PHEM and incubated in the dark for 2 h at RT with the following secondary antibodies from Life Technology (Waltham, MA USA): goat anti-mouse IgG1 coupled to Alexa Fluor 546 to detect HBZ, goat anti-mouse IgG2a conjugated to Alexa Fluor 488 to detect Tax-1, and goat anti-rabbit IgG conjugated to Alexa Fluor 488 or to Alexa Fluor 546 to detect vimentin, CD4 or CD19. For co-staining with directly labelled antibodies, after extensive washing with 1x PHEM, anti-CD8 rabbit monoclonal antibody directly conjugated to Alexa Fluor 647 (clone EP1150Y, ABCAM) and mouse anti-human CD25 monoclonal antibody directly conjugated to Alexa Fluor 488 (clone BC96, BioLegend) were added after the indirect immunofluorescence for two hours at room temperature. Similarly, after indirect immunofluorescence, the nuclei were stained by incubating the cells with DRAQ5Fluorescent Probe (Thermo Scientific, Waltham, MA USA), for 30 min at room temperature. After washing, the slides were mounted on coverslips with the Fluor Save reagent (Calbiochem, Vimodrone (MI), Italy) and examined by a confocal laser scanning microscope (Leica TCS SP5; HCX PL APO objective lenses, 63x original magnification, numerical aperture 1.25). Images were acquired and analyzed by LAS AF lite Image (Leica Microsystem, Milan, Italy) and/or Fiji (Image J) softwares.
